# Feasibility and Engagement of Multi-domain Cognitive Training in Community-Dwelling Healthy Elderly in Shanghai

**DOI:** 10.3389/fpsyt.2021.781050

**Published:** 2022-01-26

**Authors:** Ze Yu, Xia Wu, Rui Jiang, You Chen, Yuan Shen, Chunbo Li, Wei Feng

**Affiliations:** ^1^Department of Psychological Medicine, Fudan University Shanghai Cancer Center, Shanghai, China; ^2^Department of Oncology, Shanghai Medical College, Fudan University, Shanghai, China; ^3^Department of Endocrinology, Jing'an District Centre Hospital of Shanghai (Huashan Hospital Fudan University Jing'an Branch), Shanghai, China; ^4^Qingdao Mental Health Center, Qingdao University, Qingdao, China; ^5^Shanghai Yangpu District Mental Health Center, Shanghai, China; ^6^Department of Psychiatry, Tenth People's Hospital, Tongji University, Shanghai, China; ^7^Shanghai Key Laboratory of Psychotic Disorders, Shanghai Mental Health Center, Shanghai Jiao Tong University School of Medicine, Shanghai, China

**Keywords:** healthy elderly, multi-domain cognitive training, feasibility, engagement, community

## Abstract

**Background:**

In recent years, cognitive training has been one of the important non-pharmaceutical treatment methods that could delay cognitive decline and improve quality of life in the elderly. In different types of cognitive training, both the cognitive domains focused on and their training methods widely vary. This study aimed to explore the feasibility and engagement of multidomain cognitive training in Chinese community-dwelling healthy elderly.

**Methods:**

Based on the cluster sampling method, a total of 151 healthy elderly, aged 70 or above, who lived in the neighborhoods in Shanghai met the inclusion criteria and agreed to participate in the study. Among them, 90 participants were assigned to the cognitive training group (intervention group), and 61 were assigned to the no cognitive training group (control group). Participants in the intervention group attended a 1-h multidomain cognitive training course twice a week for 12 weeks (total of 24 times), which targeted memory, reasoning, strategy-based problem-solving skills, etc. The control group did not receive any intervention.

**Results:**

There was a significant increase in test scores of story recall (*t* = −8.61, *p* = 0.00) and Raven's Standard Progressive Matrices (RSPM) (*t* = −10.60, *p* = 0.00) after in-class interventions. The overall completion of homework was 77.78%. Fifty percent of the 90 participants completed self-training. The top three self-training methods were physical exercise, reading books and newspapers, and watching TV. The overall attendance rate of the intervention group was 76.14%, and more than 50% of them had an attendance rate of 77.8%. The attendance rate was positively correlated with years of education and baseline SF-36 (physical functioning, general health, vitality, and mental health) scores, whereas it was negatively correlated with baseline disease index and fatalism of personality indicators (*p* < 0.05).

**Conclusion:**

The elderly with higher educational levels and better physical and psychological conditions had a higher engagement in multidomain cognitive training. The training course proved to be rational, feasible, and effective for community-scale application.

## Introduction

As one of the non-pharmacological intervention methods, cognitive training results in the efficient improvement of cognitive abilities in the elderly (1, 2). Among various methods of cognitive intervention, two methods are widely practiced, namely, single-domain and multidomain cognitive training. The single-domain cognitive training is applied to non-demented elderly subjects for improving single cognitive function. In this regard, the largest multicenter, randomized, single-blinded intervention study involving 2,832 elderly subjects is the “ACTIVE” (Advanced Cognitive Training for Independent and Vital Elderly Trial), which was launched in 1998 in the United States. The subjects were given single-domain cognitive training on memory, reasoning, and thought processing speed 10 times. Thereafter, four times of booster training were provided 1 year later, and follow-up was conducted at 2, 5, and 10 years after intervention. The results show that the benefits of memory training lasted for 5 years, whereas that of reasoning and thought processing speed lasted for 10 years (3). The other cognitive training method involves the integration of single-domain training to construct multidomain training methods, e.g., integration of social ecological and working memory task, strategy and reasoning training, processing speed training, puzzles, and fitness training (4, 5) to achieve the best training effect in each cognitive domain with widespread impacts (6).

Because most of Chinese elderly care more about their physical condition, cognitive training is not well accepted. Single-domain cognitive training had a higher shedding rate and more limited cognitive improvement. In the past 10 years, we have designed multiple cognitive training contents.

The effects of our cognitive training are reported in previous published papers, including a 5-year follow-up study of multidomain cognitive training for healthy elderly community members, mechanisms in brain imaging, and associations between gene polymorphism and intervention efficacy (7–10). As a continuation of our previous findings, this is the first time that we report the contents as well as feasibility and engagement of multidomain cognitive training methods in elderly from the Chinese community.

Elderly people were given 12 weeks of daily self-training and multidomain in-class cognitive training, including a story recall test, associative vocabulary memory (AVM), irrelevant vocabulary memory (IVM), and Raven's standard progressive matrices (RSPM), etc.

The control group did not receive any class training or intervention. Cognitive assessment was performed on the control group at baseline, at the end of intervention, 6 months after intervention, and 1 year after intervention, including Stroop, NTBE etc. These results are reported in our previous paper, too (7–10).

## Materials and Methods

### Participants

A total of 347 elderly subjects were screened by cluster sampling in a community in Shanghai. The cluster sampling unit was the community under the jurisdiction of the residents' committee.

### Inclusion Criteria

In this study, subjects were selected based on the following inclusion criteria: (1) age over 70 years, (2) no history of serious somatic disease, (3) no history of neurological and neuropsychiatric disorders, (4) are sufficiently educated, and (5) no history of severe vision or hearing loss. Enrollment was conducted by two psychiatrists in the community. One psychiatrist was responsible for the enrollment of the intervention group and another for the control group. Every 50 elderly persons were evaluated by one psychiatrist. All participants signed informed consent.

### Baseline

The baseline health status of the participants was assessed based on their demographic data, disease index, 36-item Short-Form Health Survey (SF-36) (11, 12), personality indicators (13), and neuropsychological tests, etc.

### Multidomain Cognitive Training

#### Face-to-Face Session

Form of course: Two psychiatrists conducted 1-h face-to-face, multidomain cognitive training to the elderly in the intervention group twice a week for 12 weeks (total of 24 times). In-class tests were performed as part of the course.

Course arrangement: Story recall training (three times), AVM (twice), IVM (twice), RSPM (four times), face and name training (twice), strategy training (twice), learning training to use a map of Shanghai (twice), handicraft making (twice), calligraphy and painting (three times), and fitness exercises (twice). Each training session focused on one method, described below:

1. Story recall training: The story content was derived from the comprehension and memory section of the revised Chinese version of the Wechsler Memory Scale (WMS), which had two parallel versions. Each version included three stories (A, B, and C) of which B and C were recorded in advance. While the recording of a story was played, participants were presented a slideshow of that story instead of reading or showing the story cards to them. After that, participants were asked to write down the story in detail. Scores were given according to the WMS operation manual. The final score was the sum of the two test scores divided by two. A higher score indicated better memory. As participants were all over 70 years old and the results were only used for intragroup comparison, we did not convert the scores into standard scores. Then, participants were trained on the spatial-mnemonic method. The training method relied on the fact that visualization of the story plot in the mind could better improve the memory recall capacity instead of just memorizing words.
2. Face and name training (14, 15): Participants were presented with 12 slides composed of faces and names, each lasting 30 s. After that, participants were randomly presented with slides that contained only faces and were asked to write the correct names to test their memory and association abilities. Faces and names were searched and downloaded from the Internet, and images were formatted into uniform size, pixel, and background. Twelve faces were randomly distributed into three groups, of which two groups were used for testing while the other group was used for practice. The sum of the correct numbers was the score. Higher scores reflected better daily working memory, association ability, and ability to use strategies. Participants were then trained to associate names with facial features in the spatial representation mnemonic method. When memorizing names, the participants were trained to convert them into memorable words based on homophonic sounds.
3. AVM: The test content was derived from two sets of tests (10 pairs of vocabulary in each set) in association with learning, according to the Chinese revision of WMS. Those vocabularies were made into recordings and slides, which were played at the same time during the test. Five sec after the end of the play, one vocabulary from each pair was played again using the same recording and slide. The order of the presentation was according to the scale. Participants were trained on associative memory skills. In combination with the inner association of each pair of vocabularies, they used helpful methods, such as sensory and categorization association, to memorize. For example, if the words are “east” and “west,” their associations are both directions, and if the words are “glasses” and “water,” their associations are both colorless and transparent. The more associations the participants made for each vocabulary, the more impressed they were with the words they needed to remember. Recordings and slides were performed three times in different orders of presentation. Associative vocabularies were divided into two categories: easy and difficult. The participant earned one point for each correct word. The scores of easy vocabularies in the three tests were added up and divided by two, and the scores of difficult vocabularies were also added up. The sum of these two scores was then used as the total score of this test. Scores range from 0 to 21 points.
4. IVM: Test vocabulary was derived from two sets of commonly used vocabularies (each set contained 15 vocabularies) in the Rey Auditory Verbal Learning Test (AVLT), which were prerecorded at a rate of one per second. These recordings were played to the participants, and they were asked to recall as soon as the play stopped. Participants were trained on reduced utilization of their memory units and improved memory effect through strategy training, such as sentence making and classification (15, 16). For example, if the 15 words we used were drum, curtain, doorbell, coffee, school, father, moon, park, hat, farmer, nose, hen, color, house, and river, when using the classification method, participants memorized similar words together, such as park and school, house and curtain, farm and hen. It could reduce the number of memory units. Another method is sentence making. In this way, participants made sentences to transform the irrelevant words into concrete and meaningful sentence. For example, his father is a farmer, wearing a hat and living in a house. There is a school on the left, a park on the right, a river in front, and a hen at home. He was sniffing coffee at home, and when the drum bell rang, he pulled open the curtains to let in the moon. The participant earned one point for each correct word. Scores range from 0 to 15 points.
5. RSPM: The test content was derived from the SPM. The Chinese version of the RSPM was revised by the National Revision Collaborative Group (Professor Zhang Houcan et al.) in 1985 (17). It is purely a non-verbal and progressively harder intelligence test consisting of 60 pictures with groups A, B, C, D, and E. Group A tests perceptual discrimination, graphic comparison, graphic imagination abilities, etc. Group B tests similar comparison, graphics combination abilities, etc. Group C tests comparative reasoning and graphic combination abilities, etc. Group D tests series relationship, graphic fitting abilities, etc. Group E tests abstract reasoning abilities, such as interchangeability and interleaving. Each test question consists of a large picture with a missing part and six to eight smaller pictures as options. In the test, participants were asked to figure out which of the smaller pictures was the best fit based on the correlation between the images in the larger picture, which was mainly used for the intelligence assessment. The participant earned one point for each correct question. The total score was 30 points. The higher the test score, the better the discriminating, comparative reasoning, serial relation, and abstract reasoning abilities. Researchers explained to the participants how to solve the problem, and then participants discussed problem solving methods with each other in class.
6. Processing speed (learning to use a map of Shanghai): In a certain period, participants found a specified target according to the road and bus index. The higher the score, the better the ability to understand, use skills, and processing speed. Researchers explained to the participants about the searching method, and then participants discussed the searching method with each other in class. For this part, each test was two points and the total score was 10 points.
7. Strategy training: In this session, the researchers summarized a series of problem-solving strategies, such as the spatial representation mnemonic, classification, and sentence-making methods (15, 18, 19). They reguided the elderly to sort out how to apply each strategy pertinently in the set-specific scene.
8. Puzzle and fitness training: This session consisted of two parts. One kind were handicraft making silk stockings flowers, such as simulated calla lily and crabapple flower, calligraphy writing skills, coloring and drawing training. Another was fitness exercise, including teaching elderly about fitness precautions and aerobics for the aged. Part of this aimed to broaden the hobbies of the elderly and taught them fitness tips.

#### Homework

The intervention group completed one homework assignment after each two training sessions, including reading passages and answering questions, calligraphy, painting, etc.

#### Self-Trainings

Combined with personal interests, self-training at home included physical exercise, playing chess and cards, writing, daily life skills, and sensory training, etc. Participants recorded daily training content, duration, and training effects and made a regular self-summary themselves (18).

### Statistical Analysis

The original data were scientifically coded according to the principles of statistics. Epidata3.1 was used to establish the database, and SPSS24.0 was used to perform statistical analysis. Descriptive statistical analysis, Chi-square test, *t*-test, analysis of covariance (ANCOVA), repeated measure analysis of variance, and correlation analysis were used for the statistical analyses of the data.

## Results

### Demographic Data

At baseline, 90 elderly people in the intervention group and 61 in the control group were enrolled. There were no statistically significant differences in gender, age, and educational level between the two groups (Table 1). At the end of the intervention, 83 elderly people in the intervention group and 51 in the control group were followed up.

**Table 1:** Baseline demographic data.

	**Education years (%)**				**Gender (%)**		**Age ($\bar{x}$ ± *s*)**
	** <6**	**6-9**	**9-12**	**> 12**	**Male**	**Female**	
Intervention (N = 90)	30 (33.3)	22 (24.4)	23 (25.6)	15 (16.7)	53 (58.9)	37 (41.1)	74.7 ± 3.7
Control (N = 61)	22 (36.1)	13 (21.3)	17(27.9)	9 (14.8)	30 (49.2)	31 (50.8)	75.0 ± 3.8
Statistical value	0.39^a^				1.38^a^		−0.48^b^
*P*	0.94				0.32		0.63

a *, chi-square value*;

b *, t-value*.

### In-class Test Outcomes

A total of 12 in-class tests were conducted during the cognitive training period, including story recall, AVM, IVM, RSPM, face and name training, and map of Shanghai test with an average completion rate of 96.44% (Table 2). By using the paired *t*-test, we found statistically significant differences in story recall and RSPM before and after training (*p* < 0.05). Scores of these two tests were improved after training, and the mean scores of the other four tests were also improved to varying extents (Table 3).

**Table 2:** Multidomain cognitive training in-class test results.

	**SR1**	**SR2**	**AV1**	**AV2**	**IV1**	**IV2**	**FN1**	**FN2**	**RSPM1**	**RSPM2**	**PS1**	**PS2**
Attendance	87	78	76	72	72	74	75	69	67	63	59	65
Completion	81	72	75	71	66	68	74	67	67	63	58	63
Completion rate	93.1	92.3	98.7	98.6	91.7	91.9	98.7	97.1	100.0	100.0	98.3	96.9

*SR, Story recall; AV, Associative vocabulary; IV, Irrelevant vocabulary; FN, Face and name; RSPM, Raven's Standard Progressive Matrices. PS, Processing speed*.

**Table 3:** Scores of in-class tests.

	**Baseline**		**Post-intervention**		***t***	***P***
	*** $\bar{X}$ ***	***s***	*** $\bar{X}$ ***	***s***		
SR	10.09	3.29	13.60	5.12	−8.61	**0.00** ^**^
AV	12.24	4.77	12.28	4.08	−0.09	0.93
FN	1.70	1.82	1.84	1.77	−0.60	0.55
IV	3.52	1.86	3.93	2.05	−1.61	0.11
RSPM	10.35	5.10	15.54	5.70	−10.60	**0.00** ^**^
PS	5.96	3.33	6.28	3.06	−0.90	0.37

*SR, Story recall; AV, Associative vocabulary; IV, Irrelevant vocabulary; FN, Face and name; RSPM, Raven's Standard Progressive Matrices; PS, Processing speed. Raw*

** *p < 0.01*.

### Homework

After the face-to-face multidomain cognitive training session every 2 weeks, homework was assigned 12 times, including reading training (five times), calligraphy training (four times), and painting training (three times). The number of people who completed homework is shown in Table 4. The overall completion of homework was 77.78%.

**Table 4:** Homework completion.

**Week**	**No.1**	**No.2**	**No.3**	**No.4**	**No.5**	**No.6**	**No.7**	**No.8**	**No.9**	**No.10**	**No.11**	**No.12**
Homework	Read	Paint	Read	Calligraphy	Read	Calligraphy	Paint	Read	Calligraphy	Paint	Read	Calligraphy
Completion Number	80	82	80	75	74	74	67	58	64	63	60	63

### Self-Training

At the beginning of multidomain cognitive training, self-training tasks were assigned mainly based on the participant's own interests. Self-training instruction manuals and recorded forms were provided to each of the participants. A total of 45 valid records were received at the end of the 12-week training program, accounting for 50% of the 90 participants. According to daily records, the descending order of training content in accordance with the complete number of participants was physical exercise, reading books and newspapers, watching TV, playing chess and cards, calligraphy, listening to music, hand-knitting, singing, and dancing, listening to the radio, using the computer, etc. (Table 5).

**Table 5:** Self-training engagement.

	**PE**	**R**	**WT**	**CC**	**C**	**M**	**SD**	**HW**	**LR**	**CU**
Number	36	34	25	11	9	8	8	6	4	2

*PE, Physical exercise; R, Reading books or newspaper; WT, Watching TV; CC, Playing chess and cards; C, Calligraphy; M, Listening to music; SD, Singing and dancing; HW, Hand-woven; LR, Listening to the radio; CU, Computer using*.

### Attendance and Its Influencing Factors

**Figure 1:**
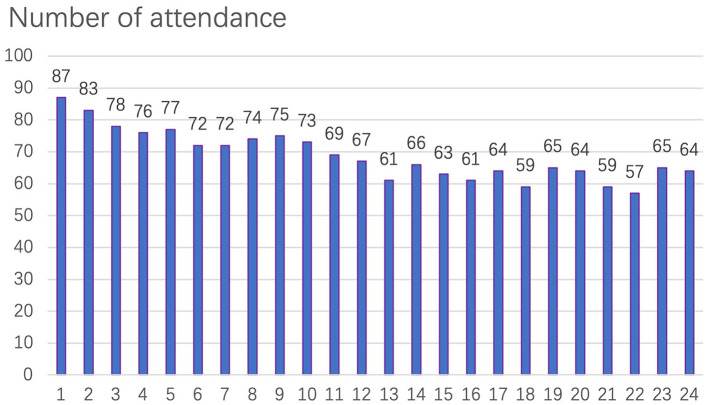
Cognitive training attendance.

The cognitive training attendance rate was calculated as (attendance times/24) ×100%. Among the 90 participants in the intervention group, 23 elderly had a 100% attendance rate. The specific attendance for each cognitive training is shown in Figure 1. The average attendance rate of the intervention group was 76.14%, and those with attendance rates over 50% accounted for 77.8% of the total number of participants (Table 6). Attendance was positively correlated with the education level, baseline SF-36 physical functioning factor score, baseline SF-36 general health factor score, baseline SF-36 vitality factor score, baseline SF-36 mental health factor score, and negatively correlated with baseline disease index, personality index fatalism factor score (*p* < 0.05) (Table 7).

**Table 6:** Attendance of the intervention group.

**Attendance (%)**	***p* < 25%**	**25% ≤*p* < 50%**	**50% ≤*p* < 75%**	**75% ≤*p* < 100%**	***p* = 100%**
Number (proportion)	9 (10.0%)	11 (12.2%)	9 (10.0%)	38 (42.2%)	23 (25.6%)

**Table 7:** Correlation between attendance and baseline assessment.

	**PCC**	***p***
Education level	0.235^*^	0.026
MMSE	−0.038	0.724
Disease index	−0.224^*^	0.034
PF	0.313^**^	0.003
RP	−0.018	0.870
BP	0.185	0.081
GH	0.214^*^	0.042
VT	0.391^**^	0.000
SF	0.146	0.170
RE	0.198	0.062
MH	0.220^*^	0.037
pi Extraversion	−0.158	0.136
pi Vulnerability	0.018	0.869
pi Self-efficacy	−0.077	0.471
pi Neuroticism	0.064	0.546
pi Fatalism	−0.268^*^	0.011

*Raw*

* *p < 0.05*;

** *p < 0.01. PCC, Pearson correlation coefficient*.

## Discussion

### Feasibility

This training course was an improvement and supplement to our previous training methods in which most of the elderly had difficulty in reading due to vision impairment when using the Shanghai map (20). The completion rate was improved by equipping the elderly with magnifying glasses during the training time. In the previous study, most participants reported a higher difficulty level of face and name training methods, leading to a reduced completion rate. Therefore, we adjusted the training difficulty in this study. The average scores before and after the intervention were 1.70 and 1.82 points (total score of 12 points), which were higher than those of 1.37 and 0.81 points in our previous study, but still lower than the scores of 2.3 and 4.2 points as reported in Cavallini's study (14). It might be related to the higher educational level of the elderly in their study. The average completion rate of 12 tests of in-class multidomain cognitive training was 96.44%, indicating that this cognitive training module had good operability and compliance. Because there was no requirement for writing words in Raven's reasoning test, the completion rate reached 100%, and the operability was the best. Likewise, due to more written content and higher vocabulary requirements, the completion rate of the story recall test remained low. Among the elderly, who insisted on completing this training, the effectiveness of the training was clearly reflected. In addition, some elderly reported that there were too many pictures to remember, suggesting that future studies can be performed with a lesser number of pictures.

The previous study shows that playing cards or mahjong has a significant positive effect on protecting cognitive functions in elderly individuals (21). Handicraft, calligraphy, and painting are also included in this intervention. The elderly subjects usually express strong interests with a high compliance rate, and such training items need to be added in future studies. Regarding the homework, all the elderly who completed the training rated it as moderately difficult. In future studies, homework could be set to match the training content in class so as to achieve better intervention effects.

Out of the self-training methods, most elderly subjects still opt for physical exercise, reading, and watching TV as their main training items although electronic devices are increasingly becoming popular. In this study, based on the completion rates, physical exercise ranked first. Previous studies show that physical exercise is positively correlated with cognitive abilities in healthy elderly (22–24). Both elderly subjects who follow routine exercise all their life (25) or participate only at the later stage (26) can improve their cognitive function. A study involving the Chinese elderly community shows that long-term adherence to square dancing is also beneficial to improve mild cognitive impairment (27). In the future, physical exercise–associated cognitive training should be promoted to improve cognitive function in aged individuals.

### Engagement

Previous studies suggest that a sensitive age of cognitive decline may be between 71 and 75 years of age (28–30). Thus, we selected elderly subjects over 70 years of age for the intervention group. In other studies, the attendance rate of single-domain training has been reported as 67% (18), whereas that in our previous study was 72.05% (19). After summing up the previous research experiences, we adopted the method of multidomain cognitive training and adjusted the training content to improve the learning interest of the elderly. Through health education, recreational activities, gifts, and other methods, the overall attendance rate of the intervention group was improved to 76.14%. The decrease in attendance rate in the later stage might be attributed to the cold weather condition and upcoming Spring Festival engagements. Therefore, it can be suggested to arrange the training sessions avoiding the seasons of inclement weather conditions and traditional festivities to obtain maximum attendance rate in future large-scale studies.

The correlation analysis of attendance rates showed positive correlations between higher attendance rates and higher education level, the more positive fatalism, and better physical and mental health conditions. Jessica et al. (31) found in a large sample study that education level is one of the major predictors of cognitive intervention effects. Consistently, the attendance rate was positively correlated with education level in this study, suggesting that education level might be a potential factor affecting the cognitive training outcomes. The elderly subjects having low education levels were comparatively slow in understanding the intervention method and consequently had poor test scores, which affected their confidence level and interests in learning. Therefore, their attendance was significantly decreased, and some of them even dropped out at a later stage. The elderly subjects with poor physical or mental health conditions also had lower attendance rates due to illness or mobility problems. Hence, those with more positive fatalism were more likely to receive cognitive training at the individual level.

Our previous study shows that the cognitive function of the intervention group was superior to that of the control group after multidomain cognitive training, indicating that participants could benefit from cognitive training. Therefore, it is more important to encourage the elderly with less education and poorer physical and mental health to participate in cognitive training (8). For example, we can promote the benefits of cognitive training through more popular science. The effectiveness of cognitive training combined with aerobic exercise training is also reported for elderly in poor body health. For the elderly with poor mental health, we can combine cognitive training with psychological intervention to increase their participation in class to achieve better training effects.

In future studies, we should consider the inhibitory effects in elderly subjects with low education levels. We should also modify the training strategies to improve participation rates by decreasing the difficulty level of intervention methods and grouping the subjects by comparable education levels. The use of intelligent intervention devices for online assessment, such as immersive 3-D virtual reality games, is shown to improve cognition by affecting discrimination, attention, and processing speed (32). In the recent study, we develop an APP named “Adaptive Cognitive Evaluation- Chinese Version” (33), which could implement cognitive training while reducing travel time for elderly subjects with mobility disabilities. Training compliances also need to be improved by including cognitive-behavioral therapy (CBT) for those with negative fatalism of personality indicators in future studies.

In this study, considering the feasibility and engagement of participants, we selected multiple cognitive domains to design multidimensional cognitive interventions, including memory, reasoning, and strategy training. The engagement of the elderly was significantly better than that of the single-domain cognitive training (9). In future studies, we hope to focus on the cognitive domain of each subject's cognitive impairment, so that personalized comprehensive training may lead to better engagement and more targeted and effective results.

### Strengths and Limitations

As in several other studies, there are also certain pros and cons in this study, which should be considered when interpreting the results.

This study comprehensively explored a set of cognitive intervention methods suitable for community-dwelling healthy elderly in China. Multidomain cognitive training has the capacity to cover different cognitive domains with higher dimensions and more comprehensive intervention effects. Moreover, the face-to-face cognitive training mode had good interaction, thus suitable to enhance interpersonal skills and increase social network.

The study limitations include the high average age of the participants (over 70 years old), whose attendance rates could be affected by various underlying factors, such as bad weather and sudden physical deterioration. In addition, the face-to-face training method requires a higher education level and better physical condition, both of which were compromised to a certain extent in this study.

## Conclusions

Multidomain cognitive training courses proved to be rational, acceptable, and effective in improving cognitive functions in the elderly population. The elderly with higher educational levels, better physical and psychological conditions had higher engagement during the training course.

## Data Availability Statement

The original contributions presented in the study are included in the article/supplementary files, further inquiries can be directed to the corresponding author/s.

## Ethics Statement

The studies involving human participants were reviewed and approved by Ethics Committee of Tongji Hospital of Tongji University (Approval # LL (H)-09-04). The patients/participants provided their written informed consent to participate in this study.

## Author Contributions

WF and ZY: study conception and design. WF: administrative support. ZY and XW: data analysis and interpretation. All authors have read, approved the final manuscript, collection and assembly of data, and provision of study.

## Funding

This work was supported by the National Key R&D Program of China (2018YFC1314700); the National Nature Science Foundation of China (81371505, 81200831, and 30770769); the Nature Science Foundation of Shanghai (17ZR1426400).

## Conflict of Interest

The authors declare that the research was conducted in the absence of any commercial or financial relationships that could be construed as a potential conflict of interest.

## Publisher's Note

All claims expressed in this article are solely those of the authors and do not necessarily represent those of their affiliated organizations, or those of the publisher, the editors and the reviewers. Any product that may be evaluated in this article, or claim that may be made by its manufacturer, is not guaranteed or endorsed by the publisher.
